# Transplantation of human embryonic stem cell-derived retinal pigment epithelial cells (MA09-hRPE) in macular degeneration

**DOI:** 10.1038/s41536-019-0081-8

**Published:** 2019-08-27

**Authors:** Tina Guanting Qiu

**Affiliations:** Ophthalmic Therapeutic Innovation, Boston, MA USA

**Keywords:** Embryonic stem cells, Translational research, Embryonic germ cells

## Abstract

The use of human embryonic stem cell (hESC)-derived Retinal Pigment Epithelium (RPE) transplants has advanced dramatically in different forms for clinical application in macular degeneration. This review focuses on the first generation of hESC-RPE cell line, named as “MA09-hRPE” by Astellas Institute of Regenerative Medicine (AIRM), and its therapeutic application in human, which evaluated the safety and efficacy of MA09-hRPE cell line transplanted in patients with macular degeneration. This project marks the first milestone in overcoming ethical hurdles and oncogenic safety concerns associated with the use of an embryonic stem cell-derived line. Through in-depth, evidence-based analysis of the MA09-hRPE cell line, along with other hESC-RPE cell lines, this review aims to draw attention to the key technical challenges pertinent to the generation of a biologically competent hESC-RPE cell line and distill the four key prognostic factors residing in the host retina, which concurrently determine the outcomes of clinical efficacy and visual benefits. Given that the technology is still at its infancy for human use, a new clinical regulatory path could aid in cell line validation through small cohort, adaptive clinical trials to accelerate product development toward commercialization. These strategic insights will be invaluable to help both academia and industry, collaboratively shorten the steep learning curve, and reduce large development expenditures spent on unnecessary lengthy clinical trials.

## Introduction

Since James Thompson’s first human embryonic stem cell (hESC) line created in 1998, the use of hESC lines and their derivatives for non-therapeutic and therapeutic purposes has entered into a new era of modern medicine innovation.^1^ hESC-derived cell therapies have received strict scrutiny of ethical considerations, regulatory oversight, and biological safety regarding the therapeutic applications in humans. The Geron Corporation terminated the first hESC-derived cell therapy for spinal cord injury in 2011,^2^ and Advanced Cell Technology Inc. (ACT Inc, now Astellas Institute of Regenerative Medicine, AIRM) became the leader, pioneering the first hESC-derived Retinal Pigment Epithelium (RPE) clinical investigation for retinal degenerative diseases, including Stargardts Macular Degeneration (SMD) and Geographic Atrophy (GA), secondary to Age-related Macular Degeneration (AMD). There were three key clinical trials at Phase1/2 in parallel, which tested the first generation hESC-RPE cell line (named as “MA09-hRPE”) in suspension form. These trials were started in April 2011 and completed in 2017 at several world-leading eye centers in the United States, United Kingdom, and South Korea (Clinicaltrial.gov identifiers: UK-SMD: NCT01469832; US-SMD: NCT01345006; US-AMD: NCT01344993). The United States and South Korea teams have published periodic reports, which provided early insights into the therapeutic benefits and biological behaviors pertinent to the MA09-hRPE cell line in severely-damaged subretinal microenvironment in patients with advanced AMD, and SMD.^3–6^ The most recent Phase1/2 United Kingdom SMD study report has been the most recent study in the series and has been the most comprehensive Phase ½ clinical investigations of hRPE transplant thus far.^7^ The clinical data suggested a pattern of hRPE-host interaction and cellular behaviors over the 12-month follow-up and also served as the most reliable scientific building block to further the optimization of next generation donor cell line.

This review highlights the key technical challenges pertinent to the generation of biologically competent human RPE line from hESCs, prognostic factors in the host retina, the biological safety of the MA09-hRPE cell line. Also it suggests a new clinical regulatory path for validating hESC-derived RPE cell line through small cohort, adaptive clinical trials.

## Strategic insights on clinical safety and efficacy of hESC-RPE transplant (Phase ½ studies)

When the first MA09-hRPE line was tested in patients (2011), there were controversial debates regarding the ethical and oncogenic safety concerns about using an hESC line in human.^8,9^ Since then, a relatively large Phase 1/2 clinical cohort of patients (*N* = 38) has received MA09-hRPE suspension transplantation, and the MA09-hRPE line has demonstrated a good clinical safety profile^3–7^. Other variations of the hRPE transplant such as hESC-RPE in suspension (Lineage Cell Therapeutics Inc.^10^), hRPE on synthetic membrane patch (Santen Inc.^11,12^ and University College London (UCL)/ex-Pfizer^13^) and induced Pluripotent Stem Cell (iPSC)-derived hRPE in either suspension or monolayer-sheet (Riken Institute^14^) have been reported with good clinical safety with no tumorigenic concerns, although each study had less than 10 patients. The risk of undergoing uncontrolled proliferation after transplant is reduced with hESC-RPE lines because these cells are fully differentiated and restricted to their lineage. Further, the subretinal microenvironment in adult mammals appears to possess suppressive or inhibitory cues that prevent neural lineage cell proliferation.^15–17^ For example, rat embryonic day-17 derived photoreceptor progenitor cells can quickly switch off mitogenic signals, committing to post-mitotic fate within a week upon transplantation in the subretinal space in retinal degenerative rat model.^15,16^

Clinical safety studies of the MA09-hRPE line found no clinically significant retinal vasculitis, retinitis, tissue necrosis, cystoid macular edema, or hemorrhagic inflammatory response caused by the hRPE grafts. Therefore, there is no clinically measurable evidence indicating that the hRPE grafts cause any acute or chronic immune-inflammation. Graft-host immunological rejection has been well-controlled with immunosuppressants and steroids. Nevertheless, an early withdrawal of immunosuppressant and steroids could trigger a local immune-response, especially when the blood–retinal barrier was disrupted by the subretinal injection. Even if this is a minimally invasive procedure, there is still a risk of a needle scratch to the host RPE layer around the retinotomy site. For example, the South Korea study team reported that one patient had a mild build up of subretinal fluids around the injection site.^5^ This patient stopped the treatment of immunosuppressants at post-transplant week 4 instead of continuing drug treatments for 6 weeks or longer.^5^ Also, a tiny host RPE window defect was evident around the retinotomy site, which was in line with the needle entry, a point of contact scratch, and suggested a possible disruption of blood–retinal barrier.^5^ Graft depigmentation has been seen in a few patients (patient 9 and 4 at post-operative month 6 and 12, respectively) in the UK-SMD report.^7^ This may be due to immune-rejection; however, the disappearance or reduction of hRPE cell pigmentation could also be a result of host retinal remodeling and Muller glia phagocytosis, which engulfs the grafting hRPE cells. In advanced retinal degeneration, as seen in this patient cohort, macrophages might be recruited for eliminating the dead cell debris. This process is different from classic antigen–antibody reaction or humorous immune-rejection. Like tissue organ transplant, the graft survival is a prerequisite for hRPE functionality in the host subretinal space and represents the most formidable technical challenge. Although classic immune-rejection in immune-privileged subretinal space is clinically manageable, harsh host microenvironment poses the biggest threat for graft survival. These prognostic risk factors can be summarized in four key pathological processes in patients with macular degeneration: (i) Bruch’s Membrane (BM) aging, rigidity, thickening, or atrophy,^18,19^ (ii) subretinal scarring predominated by Muller glia proliferation,^20^ (iii) local chronic para-inflammation featured as imbalanced complement activities (e.g., N-retinyl-N-retinylidene ethanolmine debris),^21^ (iv) significant choroid ischemia (e.g., “dark” macula in SMD),^22,23^ which collectively or individually contribute to the dynamic challenges of graft survival in a diseased setting. So far, there are no available treatments to pre-emptively manage these factors.

Although the primary objective of Phase ½ clinical trial is to ensure patient safety, the therapeutic benefits from hESC-RPE grafts have been very encouraging. Many patients had clinically measurable visual improvements.^4–6^ In the worst scenario, graft cell-induced biological activities were clinically discernable, such as improved retinal sensitivity, or Microperimetry (MP1) changes from scattering, and unstable eccentricity shifting to the focal graft area, which was consistently found among different subjects treated in the UK-SMD study cohort (ref: the above statement is based on the raw data analysis from the Data and Safety Monitoring Board, DSMB for UK-SMD study cohort with Clinicaltrials.gov identifier: NCT01469832). One exceptional case was observed in an advanced AMD patient, who had achieved a remarkable visual improvement from 20/400 at baseline of Best Corrected Visual Acuity (BCVA) on Early Treatment Diabetic Retinopathy Study (EDTRS) to 20/40 after MA09-RPE transplant (ref: internal DSMB of US-AMD study cohort with Clinicaltrial.gov identifier: NCT01344993, pool data results were published^4^). This particular patient had a large, well-demarcated GA lesion and also had an excellent prognostic profile. Namely, the host retina had an extremely well-adapted and engorged choroidal vascular circulation underneath the macular region. There was little subretinal scar, drusen, drusenoids, accumulative debris, or lipid precipitation, and the BM line was continuous and soft on Spectral Domain-Optical Coherence Tomography (SD-OCT) with no signs of clinically significant inflammatory activities. The host neural sensory retina in GA lesion had 3–4 layers of photoreceptor cells remaining and looked transparent, elastic, and vibrantly healthy. The only thing missing in this patient was the RPE layer, which exhibited a large elongated patchy loss around the macular region. This type of patient is considered the ideal candidate for hRPE transplant.

Notably, patients enrolled in this Phase ½ study series for MA09-hRPE line test are at very advanced disease stage (baseline BCVA is less than 20/400 on EDTRS in the studied eyes), therefore the visual functionality without further deterioration over time should be considered treatment benefits. In the UK-SMD study cohort,^7^ the UK team took extra care in selecting the most advanced SMD patient population (*n* = 12), who suffered more severe pathological damage compared to the patient cohorts in the US-SMD and the US-AMD clinical trials.^3–6^ In many cases, patient’s BCVA at baseline was hand motion or counting fingers without central macular fixation, though the inclusion criteria in the UK-SMD trial were set the same as those in the US-SMD trials.^7^ The retinal structure on SD-OCT image series showed that there was hardly any photoreceptor layer left and only scarce cone or rod islands at the site selected for surgical injection (bleb).^7^ The lack of robust vision gain in the UK study was likely due to the progressed pathogenicity of the injection region and suboptimal donor cell competence.

The ultimate goal of hRPE transplants is to reconstruct the anatomic and functional integrity of the blood–retinal barrier by replacing the loss of RPE cells in diseased retina; however, the visual functional benefits observed in this UK-SMD study cohort are largely associated with the neural protective effects stimulated by grafted hRPE cells. The hRPE cells produce growth factors and cytokines through paracrine and autocrine signaling. Some cytokines such as pigment epithelium derived factor, interleukins, vascular endothelium growth factor, insulin-like growth factor, integrins, and metalloproteinases may have immune-modulation or rejuvenation effects. Similar neurotrophin and biological effects have been observed in patients with advanced AMD and SMD who were treated with MA09-hRPE in the US and South Korea clinical trials.^3,5–7^ The paracrine and autocrine secretive effects are key differential advantages of using young healthy vibrant hESC-derived hRPE cells compared to autologous RPE sheet transplant, in which aging RPE graft provides limited biological effects and visual benefit to patients.^17^

## The UK-SMD case highlight and product safety of MA09-hRPE line

This review aims to provide a deeper and broader understanding of the MA09-hRPE clinical study results and offset the safety alarm concluded at the recent UK-SMD study report^7^ (Identifier: NCT01469832). The final conclusion of this study report by the authors can be summarized as follows: “the potential for harm and indicated that intervention at earlier stage of degeneration should be approached with caution”.^7^ This statement is based on the clinical evidence of focal reduction in retinal sensitivity, corresponding to the thinning of the remaining host retina overlying the hyper-pigmented graft in patient No: 10, who was treated with the highest dose (200,000 MA09-hRPE cells).^7^ After careful examination of the figures and surgical details,^7^ in my personal opinion, this should not be taken as an alarming safety signal. The focal sensitivity reduction was not caused by intrinsic harmful molecules, pathogens or biological components derived from the MA09-hRPE donor source. In this particular individual,^7^ the graft hRPE seeding density appears to have contributed to the problem- about 80% hyper-pigmented cells overlap with the remaining host RPE layer, partly and possibly due to intrinsic cell aggregates or clusters that precipitated upon the completion of injection (inferior bleb). Of note, prior to the injection, the preparation of the hRPE suspension from a frozen vial was a key factor. Often times, the cell suspension is composed of a mixture of single cells with aggregates of 2–3 cells and occasionally 3–5 cells. If not manipulated properly, these small clusters have an inclination to aggregate and form larger cell clumps, which could cause multi-layer cell aggregates instead of a monolayer cell sheet or patch. If such a high dose is confined within a very small bleb area, such as what was observed in this patient No. 10, the incidence of forming cell clumps in the subretinal space is very likely. Post-operational supine position might also affect seeding pattern. Dosing strategies should be guided by seeding density, based on lesion size; one-size does not fit all. Therefore, this should be a matter of a surgical parameter design that needs an individualized treatment. Delivery tactics can be improved through providing a thoughtful surgical operational protocol set forth for principle surgeons.

## Key problems pertinent to hESC-RPE cell lines

One fundamental problem lies with cell biological competence and metabolic deficiency, which determines graft cell survival, in vivo functionality, and, ultimately, patient visual recovery. Many hESC-RPE cell lines suffer from various biological deficiencies, from lacking of phagocytosis (e.g., California hRPE-Patch^11,12^) to missing metabolic proteins pertinent to anti-oxidative and melanin maturation.^18,24^ Addressing the metabolic deficiencies in hESC-RPE cell lines should be the priority of next phase development taskforce. Scientists at the forefront of preclinical research should be aware that many proteins and cytokines associated with hRPE cell metabolic activities are difficult to gain through a single-cell lineage, and monolayer culture environment in a short time (4–6 months).^12^ The metabolic deficiency in hESC-derived RPEs needs to be addressed as we move forward in a stepwise fashion towards patients with better vision at baseline. Such a problem is not just for MA09-hRPE line, but also to the entire field of hESC or iPSC-derived hRPE transplant. Study groups at Riken, Japan (iPSC-hRPE sheet^12^), California US (hRPE-Patch^12,18^), UCL/ex-Pfizer (hRPE-Patch^13^), and Lineage Cell Therapeutics Inc. (hRPE suspension^10^) are newcomers and have only tested handful patients. Creating a biologically competent and scalable hRPE cell line remains to be the bottleneck technical challenge.

The fundamental challenge for a hESC-RPE line to gain a full spectrum of its biological competence lies at the short cut timeframe in current culture systems. Compared to the hRPE differentiation and maturation in a full term normal human embryonic development, the in vitro differentiation protocol from totipotent hESC to hRPE only takes 12–22 weeks (depending on protocols^12,13,25^). Morphologically, these derivatives may have gained pigmented hexagonal shape and tight conjunction. However, broad cellular functionality, which includes intact signaling pathways of anti-oxidative properties, metabolic basis of visual cycle processes, cellular adhesion, phagocytosis, and cell survival, is yet to be determined. Studies suggest that hESC-RPEs exhibit epithelium morphology but have challenge to acquire the genetic profile without an interaction with photoreceptor cells. This effect is likely due to the single-cell lineage differentiation environment in petri dish.^26^ Sugino IK and Zarbin M used BM explant culture model to study hESC-RPE cell characteristics, and they found that the hESC-RPE obtained from a single blastomore-derived ESC line (MA01) did not express metabolic proteins such as cellular retinaldehyde-binding protein, and had increased apoptosis upon seeding on an aged BM compared to native fetal RPE cells.^18^ A recent comparative proteomic analysis of hESC-derived RPE cells demonstrated that the hESC-differentiated cells only capture about 80% of the protein expression profile compared to native RPE donor cells. The metabolic protein expression profile alone was only 40% similar to native human RPE cells.^25,26^ Pathway analysis of the protein expression profile also suggested mitochondrial dysfunction and downregulation of oxidative phosphorylation in the hESC-RPE compared to native human RPE.^25,27^ Such defects of mitochondrial function present a greater challenge in iPSC compared to hESC donor sources.^24^ Nevertheless, recent studies have shed a light on the calcium ion channel functionalities and immunological properties of hESC-RPE cell lines.^28,29^ In addition, Dr. E. Bannin et al. at Lineage Cell Therapeutics Inc. reported Phase ½ interim results of their hESC-RPE (OpRegen) transplantation in patients with advanced dry AMD at 2019 Association for Research in Vision & Ophthalmology annual meeting, which showed signs of drusen reduction and improvements of the ellipsoid zone in some patients (https://www.arvo.org/annual-meeting/program/online-planner/). This is a step advance of hESC-RPE gaining additional metabolic competences such as phagocytosis, which has not been reported before. Through collective efforts, hopefully a biologically competent cell line can be generated that propels for a better graft survival and functionality in diseased settings.

A key clinical phenotypic problem of grafted hRPE cells is hyper-pigmentation. Notably, MA09-hRPE transplantation in both SMD and AMD along with recent RPE-patch transplantation in advanced AMD (California Group) observed increased pigmentation.^4–7,11,12^ The hyper-pigment changes in hRPE grafts indicated accelerated aging processes due to insufficient or lack of anti-oxidative components. One study showed that melanin is the key retinal pigment responsible for photosensitized oxidation of exogenous ascorbate.^30^ The pigment granules act as an electron transfer agent and stimulate the photo-oxidation of unsaturated fatty acids in the photoreceptor cells.^27,30^ In a recent report by Kashani AH, hyper-pigment changes in hRPE-patch (hRPE layer on synthetic basement membrane) was observed following in vivo subretinal transplants in advanced AMD patients with progressive GA lesion.^11^ A 4-year study on the MA09-RPE transplants in GA patients by Schwartz SD reported a different pattern of pigment change.^6^ The deposition of pigment granules were in clustered strings or dotted lines alongside the well-demarcated border of GA area, which underpins the most active remodeling activities, predominantly governed by Muller glial cell proliferation and scar formation with or without macrophage involvement. These clinically visible pigment granules could be a result of hRPE cell death releasing melanin granules, instead of an evidence of graft survival as previously thought.^6^ The genetic components, epigenetic profile, and protein expression profile required for hESC-RPE cells to enable a full spectrum of metabolical and biological functions upon in vivo transplantation will be the key taskforce for scientific research. Pharmacological intervention to address prognostic risk factors residing in host pathological microenvironment is equally important for the success of hRPE transplant in the long term.

## Future direction and strategic principle

**Fig. 1:**
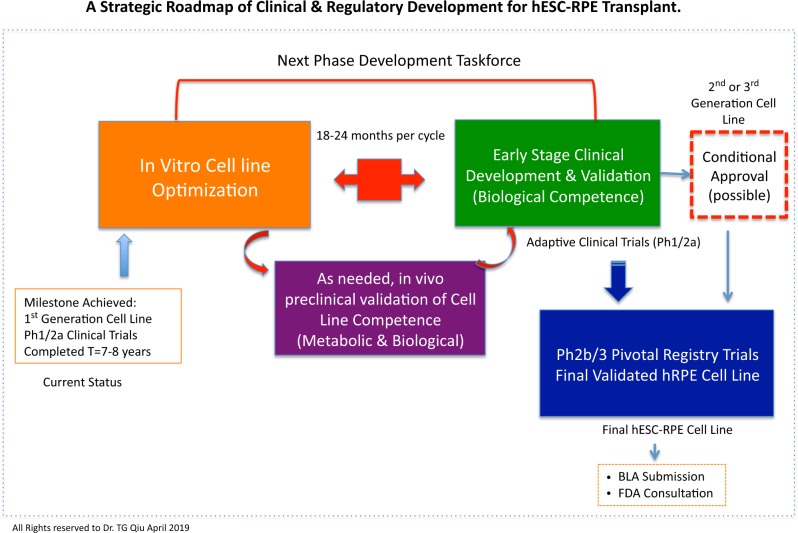
A Strategic Roadmap of Clinical & Regulatory Development for hESC-RPE Transplant. This figure provides a strategic roadmap of clinical regulatory development framework pertinent to hESC-RPE cell line optimization and validation through adaptive clinical trials in small patient cohorts. It depicts a viable and cost-effective translational path, back and forth between in vitro cell line optimization and early stage clinical trials, which has strategic importance to the development and commercialization of hESC-derived cell therapies. This roadmap entails decades of development work in order to generate an ideal or biologically competent hRPE line for global commercialization. However, an expedited or conditional approval with term-limited authorization may be possible as the second- or third-generation cell line enters into Phase 1/2 or proof of concept clinical trials, of which if the study results can predict clinical efficacy with reproducibility and confirming product safety.^33,34^

hESC and iPSC-derived cell therapies are still in their infancy, with limited human experiences (less than 100 patients receiving hESC or iPSC-derived hRPE transplant as of today). There is a steep learning curve and a huge gap to bridge between preclinical data to clinical translation.^31^ In drug development, candidate selection takes place prior to human clinical trials; whereas, in hESC-hRPE cell tissue transplant, candidate cell selection may require going back and forth between in vitro cell optimization and clinical validation through a small cohort adaptive trial in patients (Fig. 1). There are limitations associated with hRPE metabolic maturation in a single-cell linage and monolayer culture system without photoreceptor influence and underlying BM support. Also, the shortcomings of inherited retinal degeneration animal models present a unique challenge for monolayer RPE transplant, especially in small rodent eyes. Unlike in drug development, once the candidate cell line is validated with proficiency in early development, the success of late stage (Phase 2b/3) trials should be eminent as long as appropriate patients are identified. Documenting the subtle clinical morphological changes pertinent to hRPE aggregates, migration, cell survival/death, melanin granule deposition, and accelerated aging is of critical importance. Understanding graft behaviors and establishing evidence-based clinical data interpretation are the gaps in clinical translation. In particular, going back to the laboratory for cell line optimization requires broad translational expertise. Without stepwise adaptive clinical trials assessing cell biological or metabolic competence, it is unlikely to know what level or range of targeted gene profile and protein expression would be desirable and sufficient. Although it is unlikely to generate a hRPE cell line identical to native fetal hRPE derived from a full term embryonic development, this relative large, long-term transplant study series on the MA09-hRPE cell line, especially the recent UK-SMD study report, takes us a step closer to find the right candidate hRPE cell line^3–7^.

With emerging clinical data from other forms of hRPE transplant including iPSC-hRPE sheet (Riken, Japan), hESC-RPE-patch (Santen Inc. & Patch Technology LLC, and UCL/ex-Pfizer), and hESC-RPE suspension (Lineage Cell Therapeutics Inc.), we are gaining a better understanding of hRPE-host tissue interaction and grafted cell behaviors in a variety of clinical phenotypes associated with macular degeneration, including SMD, dry and wet AMD, as well as myopic macular degeneration. Nonetheless, the steep learning curve remains elusive. In order to avoid unnecessary and lengthy path-finding expenditures, one must have drug development mindset and exercise transplant principles, while defining a cost-effective clinical regulatory path forward. On the other hand, global clinical and regulatory landscape in stem cell regenerative medicine is evolving rapidly. European Medicine Agency, Japanese Pharmaceuticals and Medical Device Agency, and US Food and Drug Administration have developed regulatory framework for expedited approval and conditional approval (Fig. 1) to further advance stem cell therapeutic product development.^32–34^ As when classic gene replacement therapy first succeeded in retinal disease, it’s with great anticipation that hESC or iPSC-derived hRPE transplant may lead the breakthrough and pave a successful clinical regulatory path for others to follow. The future is full of promise yet the journey remains intriguingly challenging.

### Reporting summary

Further information on research design is available in the Nature Research Reporting Summary linked to this article.

## Supplementary information

Reporting Summary

## Supplementary information

**Supplementary Information** accompanies the paper on the *npj Regenerative Medicine* website (10.1038/s41536-019-0081-8).
